# Multistage functional nanoplatform synergizing with robust wet-adhesive scaffolds for accelerated cranial bone regeneration

**DOI:** 10.1093/rb/rbag183

**Published:** 2026-09-10

**Authors:** Xiaohua Su, Nashun Bayaer, Xi Long, Qi Li, Siying Liu, Siying Cheng, Weiquan Shen, Wenqiang Li, Yi Zhan

**Affiliations:** Clinical Research Center, The First Dongguan Affiliated Hospital, Guangdong Medical University, Dongguan 523808, China; Laboratory Animal Center, Guangdong Medical University, Dongguan 523808, China; School of Basic Medical Sciences, Inner Mongolia Medical University, Chilechuan Dairy Economic Development Zone, Hohhot 010110, China; Department of Rehabilitation Medicine, The Eighth People’s Hospital of Nanhai District, Foshan 528216, China; School of Basic Medical Sciences, Inner Mongolia Medical University, Chilechuan Dairy Economic Development Zone, Hohhot 010110, China; College of Pharmacy, Guangdong Medical University, Dongguan 523808, China; College of Medical Technology, Guangdong Medical University, Dongguan 523808, China; College of Pharmacy, Guangdong Medical University, Dongguan 523808, China; Orthopedic Center, Department of Orthopedics and Traumatology, Guangdong Traditional Medical and Sports Injury Rehabilitation Research Institute, The Affiliated Guangdong Second Provincial General Hospital of Jinan University, Guangzhou 510315, China; Department of Orthopedic, The First Dongguan Affiliated Hospital, Guangdong Medical University, Dongguan 523710, China

**Keywords:** wet adhesion, anti-inflammatory, remodeling bone microenvironment, cranial defect repair

## Abstract

The healing process in cranial defects has many challenges. The disorder of the microenvironment and the long healing period posed significant complexity to the design of hydrogel scaffolds. Here, a multistage functional repair (immunomodulation/angiogenesis/osteogenesis) nanoplatform with synergistic and stable wet adhesion hydrogel scaffold was designed and developed. Cu-etched silica porous nanoparticles loaded with Mg-epigallocatechin gallate constituted the anti-inflammatory/osteogenic nanoplatform (CuSi@MgE). Dopa-modified oxidized sodium alginate and poly(thioctic acid) were combined via a hydrophobic cross-linking strategy to form a stable wet-adhesive hydrogel scaffold (TO). This innovative construct exhibited exceptional wet adhesion enabling seamless host-bone integration and resilience to intracranial pressure fluctuations, ensuring functional stability during skull repair. The TO@CuSi@MgE hydrogel demonstrated robust reactive oxygen species scavenging, antibacterial activity and macrophage M2 polarization that collectively remodeled the osteogenic immune microenvironment. At the same time, it promoted vascular regeneration and osteogenic differentiation in a phased manner, markedly sped up the healing of skull injuries.

## Graphical abstract

**Graphical Abstract:**
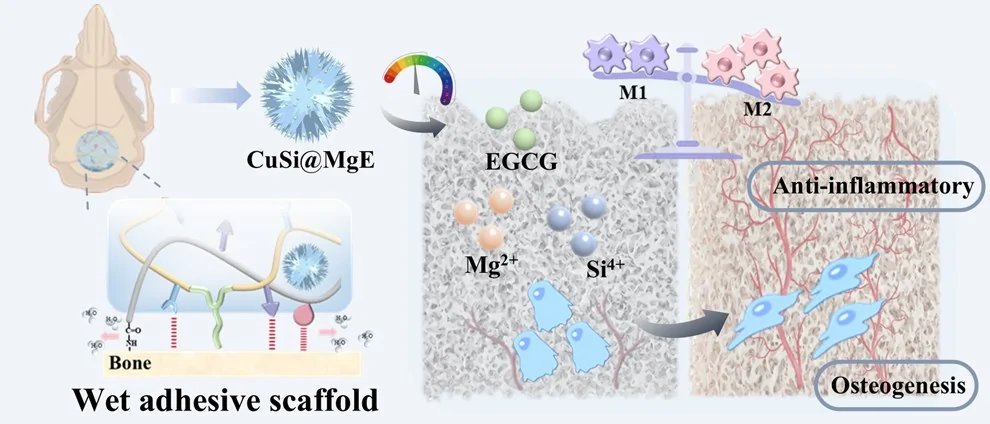

## Introduction

In clinical treatment, reconstructing cranial defects caused by trauma, osteoporosis or infection presents a substantial difficulty [1, 2]. Traditional biomaterials, such as titanium mesh and polyether ether ketone, are designed to be as inert as possible to minimize foreign body reactions [3–5]. However, their inherent drawbacks, including excessive rigidity, poor bone integration and the need for secondary surgeries, fail to yield satisfactory therapeutic outcomes [6–8]. Early in the process, excessive reactive oxygen species (ROS) can induce oxidative stress in bone defects, disrupting the growth of cells and leading to cellular senescence [9, 10]. Moreover, the traditional implant scaffolds (such as titanium mesh) may induce a rejection response after implantation, and their mismatch with the bone tissue interface tends to exacerbate the inflammatory response at the defect site, thereby impairing bone repair [11]. Therefore, the development of biomaterials that possess antioxidant and anti-inflammatory properties, capable of early modulation of the immune microenvironment and promotion of osteogenic differentiation, plays a crucial role in expediting bone defect repair [12–14].

The process of repairing skull defects involves continuous stages of immune modulation, angiogenesis, osteogenic differentiation and biomineralization [15, 16]. During the inflammatory phase, macrophages polarize into an M1 phenotype [17]. This polarization enables them to engulf provisional fibrin matrices and necrotic cells while secreting relevant factors that promote the angiogenesis of granulation tissue [18]. However, in pathological conditions, persistent M1 macrophage states lead to chronic inflammation, impeding the transition to the M2 state that triggers anti-inflammatory and repair processes, thus hindering bone repair [19, 20]. As the main polyphenol in green tea, epigallocatechin gallate (EGCG) demonstrates strong free radical-neutralizing abilities, attributed to its rich phenolic hydroxyl groups [21]. Additionally, it facilitates macrophage polarization from M1 to M2 by enhancing autophagy and relieving autophagic flux suppression [22]. Current research has demonstrated that EGCG, as a polyphenol, can form supramolecular networks through coordination with metal ions (Zn^2+^, Mg^2+^, Cu^2+^, etc.), thereby enhancing its bioavailability and reducing the toxicity of ions [23, 24]. Among these ions, Mg^2+^, with its osteoinductive properties, is widely used in bone repair therapies [25]. Furthermore, recent studies have revealed the multifaceted regulatory roles of Cu^2+^ in bone regeneration. In addition to their wide-ranging antimicrobial effects, Cu^2+^ facilitate vascular endothelial cell migration and tube formation, which plays a vital role in supplying nutrients for bone repair [26]. In summary, the rational design of metal ion-phenolic networks, combined with functional inorganic carriers, holds great promise for achieving spatiotemporal synergistic regulation of anti-inflammatory, osteogenic, angiogenic and antibacterial effects. However, a standalone metal-phenolic network can hardly be retained stably at the defect site; therefore, a stable carrier is required to achieve its sustained release and provide a scaffold for cell migration and proliferation [27, 28].

Achieving robust interfacial compatibility between the implant material and adjacent bone tissue is essential for sustaining its long-term bioactivity and therapeutic outcome [29]. Injectable hydrogels are widely employed for sealing cranial defects, with *in situ* crosslinking commonly realized via chemical routes such as photo-crosslinking, Schiff base reactions or click chemistry. Nevertheless, these methods are plagued by inherent shortcomings, including the need for external initiators, protracted curing periods and suboptimal adhesion to the host bone, which have yet to be adequately addressed [30, 31]. As an alternative, physically crosslinked adhesive hydrogels, which rely on reversible noncovalent interactions (e.g., electrostatic forces, π–π stacking, cation–π effects, metal coordination, hydrogen bonding and hydrophobic interactions) [32–35], offer a simpler and safer strategy, circumventing the use of initiators, reducing setting time and avoiding cytotoxicity associated with chemical reagents [36]. However, compared with the more straightforward approach of grafting bioadhesive groups (e.g., catechol) onto polymer chains to enhance interfacial bonding, physical crosslinking faces a critical hurdle: in wet physiological environments, a hydration layer forms at the tissue interface, which prevents intimate contact and drastically weakens adhesion [37–39].

To engineer a robust multifunctional scaffold for cranial bone defect repair, this study integrated a Mg-EGCG metal-phenolic network within Cu^2+^-etched mesoporous SiO_2_ nanoparticles, establishing a spatiotemporally controlled system that synergistically coordinates anti-inflammatory, osteogenic, angiogenic and antibacterial functions. Furthermore, thioctic acid (TA) forms poly(thioctic acid) (PTA) through the ring-opening polymerization of disulfide bonds. These chains facilitate strong hydrogen bonding with host bone tissue through abundant carboxyl side groups [40]. We introduced allyl hexanoate as a crosslinking agent to form thioether bonds with the terminal sulfhydryl groups of PTA, simultaneously introducing hydrophobic alkanes into the hydrogel network, which helps to eliminate interfacial water and expose the adhesive groups. To further enhance underwater wet adhesiveness, dopamine (DA)-grafted OSA was incorporated [oxidized alginate sodium (ODA)], whose rich hydroxyl groups and ungrafted aldehyde groups could form Schiff base reactions *in situ* with skin tissue (Figure 1). Collectively, this multimechanism adhesive hydrogel constructs a stable 3D microenvironment that not only provided mechanical support for cranial reconstruction but also establishes an immunological microenvironment conducive to osteogenesis, representing a critical step toward next-generation biomimetic bone regeneration platforms.

**Figure 1:**
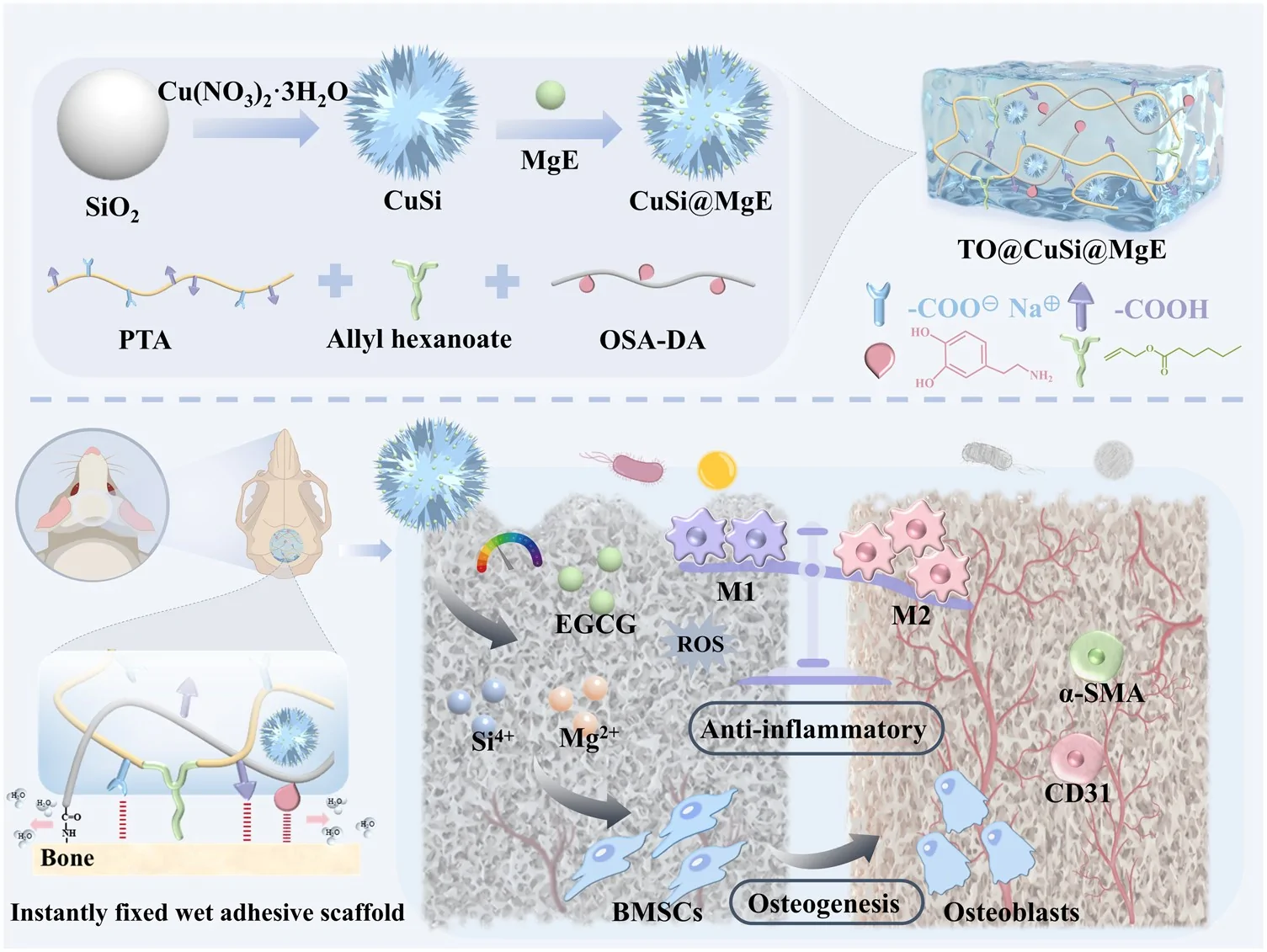
Schematic diagram of multistage functional repair nanoplatform synergizing with robust wet-adhesive polysaccharide scaffolds accelerate the repair mechanism of cranial bone defects through anti-inflammatory effects and promoting osteogenesis.

## Materials and methods

### Materials

Sodium alginate (SA, 90%, viscosity: 200 ± 20 mPa·s, *M*/*G* = 1:2, average molecular weight of 340 kDa and polydispersity index of 3.42), thioctic acid (TA, 99%), epigallocatechin gallate (EGCG, 98%), allyl hexanoate (98%), dopamine hydrochloride (98%), ethylene glycol (98%), copper nitrate trihydrate [Cu(NO_3_)_2_·3H_2_O, 99%], tetraethyl orthosilicate (TEOS, 98%), sodium bicarbonate (99.5%) and sodium periodate (99.5%) were acquired from Shanghai Macklin Biochemical Technology Co., Ltd. 1-(3-dimethylaminopropyl)-3-ethylcarbodiimide (EDC, 98%) was purchased from Shanghai Aladdin Biochemical Technology Co., Ltd. N-hydroxysuccinimide (NHS) was purchased from Shanghai Yuanye Technology Co., Ltd. Hydrochloric acid and ethyl alcohol were purchased from Tianjin Damao Chemical Reagent Factory.

### Synthesis of ODA

To synthesize OSA, 5 g of sodium alginate (SA) was dissolved in 250 mL of deionized water. Next, 5.4 g of sodium periodate was added and the solution was stirred at room temperature for 3 h under nitrogen protection while being shielded from light. After this period, 2.5 mL of ethylene glycol was added and the reaction was allowed to proceed for an additional hour. The resulting product was then dialyzed for 48 h using a 3500 Da molecular weight cutoff membrane before being lyophilized to obtain OSA [41].

Subsequently, 1 g of OSA was dissolved in 100 mL of deionized water; then 0.58 g NHS and 1.57 g EDC were added. The pH was adjust to 4.4–4.8 with 0.1 M HCl or NaOH, and the mixture was stirred for 1 h at room temperature. Next, 200 mg of dopamine hydrochloride was added, and the reaction mixture was stirred under a nitrogen atmosphere for 12 h at room temperature. Dialysis was performed using a dialysis bag (3500 Da) for 48 h, followed by lyophilization at −80 °C to yield OSA-DA (ODA) [42].

### Synthesis of CuSi@MgE nanoparticle

Initially, SiO_2_ nanoparticles were synthesized by adding 1.5 mL of tetraethyl orthosilicate (TEOS) into a 20 mL mixed solvent system of ethanol/water/NH_3_·H_2_O (5:3:1, v/v/v) under continuous stirring for 6 h at room temperature. To purify the precipitate, the initial suspension was spun at 11 000 rpm for 10 min and then washed repeatedly with ethanol and deionized water in turn. Clean SiO_2_ particles were evenly dispersed in 50 mL of deionized water. Under constant stirring, we sequentially introduced 0.7 mmol of Cu(NO_3_)_2_·3H_2_O and 5 mL of NH_3_·H_2_O. After stirring for 0.5 h, the mixture was transferred into a Teflon‑lined autoclave and kept at 140°C for 12 h under hydrothermal conditions. The final solid was collected by centrifugation, purified with three alternating ethanol/water rinses and finally dried at 60°C to give CuSi nanoparticles.

MgE nanoparticles were synthesized by reacting a blend of 5.0 mL of 3 mM MgCl_2_ and 25.0 mL of 0.5 mg/mL EGCG at 45°C for 1 h. Afterwards, 5 mL of 0.5 M NaOH was introduced into the reaction system. Following an 8h reductive process, the resulting solid was harvested via centrifugation (11 000 rpm, 10 min), rinsed with deionized water and finally freeze‑dried at −80°C to obtain the final product.

A mixture containing 5 mg MgE and 5 mg CuSi was uniformly dispersed in 10 mL deionized water with continuous stirring at ambient temperature for 3 h. The resulting hybrid nanoparticles were collected through centrifugation at 11 000 rpm for 10 min and subsequently washed, followed by −80°C lyophilization to yield MgE nanoparticles.

### Synthesis of TO@CuSi@MgE hydrogel

0.4 g of TA was dissolved in 2 mL of NaHCO_3_ (0.475 M). Subsequently, 0.05 g of OSA, 20 wt% of hexyl acrylate (relative to TA) and 0.1 wt% of CuSi@MgE nanoparticles were added, and a homogeneous pre‑hydrogel was obtained upon stirring. This pre‑gel was then irradiated under a 365 nm UV lamp for 10 min, yielding the TO@CuSi@MgE hydrogel.

### Characterization of CuSi@MgE nanoparticle

The crystal structure of CuSi@MgE nanoparticles was analyzed by X-ray powder diffraction (Bruker D8 Advance, Germany) with Cu Kα radiation (*λ* = 1.5406 Å) at 40 kV and 40 mA. The 2*θ* scanning range was set from 10° to 60°, and the scan rate was maintained at 10°/min.

The nanoparticles’ morphology, microstructure and elemental composition were analyzed using a JEOL F200 field-emission transmission electron microscope (FE-TEM, Japan) equipped with energy-dispersive X-ray spectroscopy (EDS). Prior to TEM observation, the nanoparticles were dispersed in deionized water via ultrasonication for 15 min, and a droplet of the suspension was placed on a copper grid for air-drying. Imaging was conducted at 200 kV, followed by EDS elemental mapping and point/area analysis to assess the distribution and relative concentrations of Cu, Si, Mg and other relevant elements.

### Characterization of TO@CuSi@MgE hydrogel

The structural characteristics of the material were examined by Fourier Transform Infrared Spectroscopy (FTIR, Shimadzu IR Tracer 100, Japan) and Nuclear Magnetic Resonance Hydrogen Spectroscopy (^1^H NMR, Bruker Avance NEO 600, Germany). For FTIR analysis, samples were either placed directly on the ATR crystal or prepared as potassium bromide pellets, with spectral data acquired from 4000 to 400 cm^−1^ at a resolution of 4 cm^−1^ and 32 scans per spectrum. Prior to sample measurement, background scans were recorded. ^1^H NMR spectra were obtained using a Bruker Avance NEO 600 MHz spectrometer (Germany) with deuterium oxide as the solvent.

The crystalline structure of TA was characterized by X-ray diffraction (Bruker D8 Advance, Germany), confirming successful polymerization to form PTA. For analysis, powdered samples were carefully prepared on silicon zero-background holders. Data collection was performed with a 10°/min scan rate across the 2*θ* range of 10–60°.

The morphological features of hydrogels were examined using scanning electron microscopy (Gemini SEM 360, Germany). To maintain their porous architecture, hydrogel specimens were initially flash-frozen in liquid nitrogen, followed by lyophilization. For SEM observation, the freeze-dried samples were carefully fractured to reveal internal morphology, adhered to aluminum stubs with conductive carbon tape and coated with a gold layer for enhanced conductivity. Imaging was conducted at accelerating voltages between 1.0 and 5.0 kV to achieve optimal contrast while reducing charging effects, allowing clear visualization of pore morphology and interconnectivity within the hydrogel network.

Immerse the freeze-dried hydrogel in PBS with a pH of 7.4 at 37°C and record the weight of the hydrogel at different time intervals. The swelling ratio was then calculated using the following equation:


(1) $$\mathrm{Swelling}\;\mathrm{ratio}\;(\%)=\frac{W_{t}-W_{i}}{W_{i}}\times 100\%,$$

where the *W*_i_ is the initial mass and *W*_t_ is the mass at time t.

Cylindrical hydrogel samples (diameter × height: 15 mm × 10 mm) mechanical characterization was performed on a Sunstest universal testing machine at 2 mm/min compression rate. For adhesion assessment, the material was applied to porcine skin specimens (2 cm × 4 cm) and tested at 5 mm/min tensile speed to determine shear adhesion strength. Mechanical tests were performed under controlled conditions of 25 ± 1°C and 50 ± 5% relative humidity.

Immersing the lyophilized hydrogel in PBS with a pH of 7.4 at 37°C, subsequent incubation for varying periods, and then re-lyophilizing and weighing the hydrogel to calculate the degradation rate of the material. Morphological changes in the degraded material are subsequently observed through SEM.

The release of EGCG was evaluated using a UV–Vis spectrophotometer. An EGCG standard curve was drawn. First, 0.5 g of the hydrogel material was incubated with PBS at pH 5 and 7 at different time points, respectively, and the absorbance at 278 nm was measured.

### Antibacterial and antioxidant experiments of hydrogels

Utilizing *Staphylococcus aureus* (*S. aureus*) and *Escherichia coli* (*E. coli*), the antibacterial properties of different component materials were assessed via turbidimetry and plate count methods. Initially, turbidimetry involved co-culturing 0.5 g of the material with 500 μL of bacterial suspension (10^6^ CFU/mL), followed by measuring the optical density (OD) at 620 nm over various time points using a microplate reader (BioTek Synergy H1) to generate the bacterial growth curve. Subsequently, 100 μL co-cultured bacterial suspension, which had been incubated for 18 h, was diluted 10^6^-fold. Colonies were generated in a 37°C incubator using a coating plate method, and the bacterial survival ratio was calculated using the following equation:


(2) $$\mathrm{Bacterial}\;\mathrm{survival}\;\mathrm{ratio}\;(\%)=\frac{N_{\mathrm{hydrogel}}}{N_{\mathrm{blank}}}\times 100\%.$$

In this equation, *N*_hydrogel_ represents the colony count from plates treated with hydrogel, while *N*_blank_ denotes the colony number in control group plates.

Assess the free radical neutralization potential of the materials through DPPH (1,1-diphenyl-2-picrylhydrazyl) and ABTS [2,2'-Azinobis-(3-ethylbenzthiazoline-6-sulphonate)] assay kits. Specifically, 1.8 mL of DPPH and ABTS diluent were added to different materials of 200 mg, respectively, and then incubated for half an hour. Obtain the absorbance at 517 and 750 nm with a UV–Vis spectrophotometer, vitamin C as the Positive control group, PBS as the Negative group. Calculate the DPPH/ABTS scavenging activity according to the following equation:


(3) $$\frac{\mathrm{DPPH}}{\mathrm{ABTS}}\mathrm{scavenging}\;\mathrm{activity}\;(\%)=(\frac{A_{P}-A_{S}}{A_{P}-A_{N}})\times 100\%,$$

where *A*_s_, *A*_P_ and *A*_N_ stand for the sample’s, the positive’s and negative’s respective absorbance values at 517 and 750 nm.

The hydroxyl radical (·OH) scavenging capacity was assessed using a Fenton reaction system containing 5 mM H_2_O_2_ and 1.8 mM FeSO_4_ at 37°C. After adding the 200 mg hydrogels to the ·OH solution, the remaining radicals were allowed to react with 1.8 mM salicylic acid for 30 min. The resulting product was quantified by measuring its characteristic absorption at 510 nm.

### The immune response of TO@CuSi@MgE hydrogel *in vitro*

Biocompatibility assessment of the hydrogel was performed using MC3T3-E1 murine preosteoblasts cultured in α-MEM supplemented with 10% fetal bovine serum (FBS, Gibco) and 1% penicillin/streptomycin (HyClone). Prior to cell seeding, hydrogel disks (8 mm diameter × 2 mm thickness) were sterilized by UV irradiation (30 min per side) and equilibrated in complete culture medium for 24 h. After CCK-8 analysis (performed at 1, 3 and 7 days using kit according to manufacturer’s protocol, with absorbance measured at 450 nm using a microplate reader), cell viability was further determined by live/dead staining using 2 μM calcein-AM (green fluorescence for viable cells) and 4 μM propidium iodide (red fluorescence for dead cells; Live/Dead Viability Kit, Thermo Fisher) with 15 min incubation at 37°C in the dark, visualized with an Olympus CKX41 inverted fluorescence microscope.

Cellular morphology, actin cytoskeleton arrangement and spreading behavior were characterized using a Zeiss LSM 510 confocal microscope (Carl Zeiss, Germany). Prior to imaging, MC3T3-E1 cells cultured on hydrogels were fixed with 4% paraformaldehyde (PFA) for 15 min at room temperature (RT), permeabilized with 0.1% Triton X-100 (10 min, RT) and blocked with 1% bovine serum albumin (BSA, 30 min, RT). The actin cytoskeleton was stained with Alexa Fluor 488-conjugated phalloidin (1:200) for 1 h at RT in the dark, while nuclei were counterstained with DAPI (1 μg/mL, Sigma-Aldrich, D9542) for 5 min.

Scratch test: MC3T3-E1 cells were seeded in 24-well plates (5 × 10^4^ cells/well) and grown to 90% confluence. A 200 µL pipette tip was used to create a uniform scratch, and detached cells were removed by gentle PBS washing. Fresh serum-free medium (with/without hydrogel extracts) was added, and scratch closure was monitored at 0, 24 and 48 h.

For transwell migration, HUVECs and MC3T3-E1 (2 × 10^4^ cells) were seeded in the upper chamber, while the lower chamber contained 600 µL complete medium (10% FBS) as a chemoattractant. After 12 h at 37°C, nonmigrated cells on the upper membrane were removed with a cotton swab, and migrated cells were fixed with 4% PFA, stained with 0.1% crystal violet (15 min).

HUVECs (2 × 10^4^ cells/well) were seeded on polymerized Matrigel (50 μL/well) in a 96-well plate and cultured with control or treatment medium for 6 h, among them, the intervention group used the extract solution obtained from the treatment material after 2 weeks of treatment. Tube formation was observed under a light microscope.

Following oxidative stress induction in MC3T3-E1 cells using a freshly prepared 400 μM H_2_O_2_ for 2 h at 37°C to produce ROS, intracellular ROS levels were visualized through 10 μM DCFH-DA (2',7'-dichlorodihydrofluorescein diacetate) fluorescence staining. Cells were incubated with DCFH-DA for 30 min at 37°C in the dark, washed twice with PBS and immediately imaged using a Zeiss LSM 880 confocal microscope.

Additionally, cell viability under H_2_O_2_ exposure was quantified by live/dead dual staining. After H_2_O_2_ treatment, cells were stained with 2 μM Calcein-AM (viable cells, green) and 4 μM propidium iodide (PI, dead cells, red) for 15 min at 37°C, followed by PBS washing, visualized with an Olympus CKX41 inverted fluorescence microscope.

To examine the role of hydrogels in directing macrophage polarization, RAW264.7 cells were co-incubated with the samples, then stained with primary antibodies against CD86 (M1) and CD206 (M2), and subsequently exposed to secondary antibodies conjugated with fluorophores for 30 min. Flow cytometry (BD C6, USA) was applied to measure the fractions of each macrophage subset [43].

To assess the bone-forming ability of hydrogel-cultured MC3T3-E1 cells, we performed ALP enzymatic assays and visualized calcified nodules with alizarin red. Additionally, RT-qPCR was employed to monitor the PI3K/AKT/mTOR cascade and osteogenic gene expression (Runx-2, OPN, COL-I and OCN) [44].

### Efficacy of TO@CuSi@MgE hydrogels for *in vivo* skull reconstruction

All animal welfare procedures followed the Chinese National Standard GB/T 35892-2018 ‘Guideline for Welfare and Ethical Review of Laboratory Animal’ and were approved by the Animal Ethics Committee of Guangdong Medical University (Approval Number: GYD2503027), and all animal experiments complied with the ARRIVE guidelines. Sprague-Dawley male rats (250 ± 10 g) were allocated to four treatment groups for longitudinal defect repair assessment. Surgical creation of 5 mm calvarial defects was followed by implantation of PTA, TO or composite TO@CuSi@MgE scaffolds, with saline-treated controls. Micro-CT imaging (SkyScan 1176, Belgium) at 14 weeks provided volumetric bone repair data. Histological evaluation included H&E and Masson staining, complemented by Runx-2/OCN immunohistochemistry and CD86/CD206/CD31/α-SMA immunofluorescence [45].

### Statistical analysis

All data were expressed as mean ± standard deviation (SD) unless otherwise noted. Triplicate independent experiments were conducted for each condition, with statistical significance determined by ANOVA followed by Tukey’s *post hoc* test in SPSS version 27.0. Statistically significant differences were defined as *P < 0.05, **P < 0.01 and ***P < 0.001 values.

## Results and discussion

### Preparation and characterization CuSi@MgE nanoparticle

Using silica (SiO_2_) as a template, CuSi nanocarriers were synthesized via a hydrothermal method. TEM revealed that the CuSi components aggregated into acicular nanostructures, with elemental mapping confirming the homogeneous distribution of Cu (Figure 2A). XPS analysis identified the elemental composition of CuSi as Cu, O, C and Si (Figure 2B), further indicating that Cu existed predominantly in the +2 oxidation state (Figure 2C). XRD patterns showed that the SiO_2_ template was amorphous, whereas crystalline CuO phases were detected in CuSi (Figure 2D). Subsequently, the MgE metallophenolic network was prepared. TEM imaging demonstrated its uniform dispersion and small particle size of approximately 5 nm (Figure 2E). UV–Vis spectrophotometry confirmed the successful loading of EGCG onto both MgE and the composite CuSi@MgE nanoparticles (Figure 2F). The MgE network was then loaded into the mesopores of the CuSi nanocarriers to form the CuSi@MgE composite. N_2_ adsorption-desorption isotherms for both CuSi and CuSi@MgE exhibited Type IV behavior (Figure 2G). Notably, the N_2_ adsorption-desorption volume of CuSi@MgE decreased compared to CuSi, accompanied by a less concentrated pore size distribution (Figure 2H). Additional TEM characterization coupled with elemental mapping and EDS analysis verified the homogeneous dispersion of MgE throughout the mesoporous structure of CuSi (Figure 2I). The even distribution of Mg along with the detection of C and N elements served as conclusive evidence for successful EGCG incorporation (Figure 2J and K). Therefore, a mesoporous CuSi nanocarrier loaded with the MgE metallic phenolic network (CuSi@MgE) was successfully synthesized.

**Figure 2:**
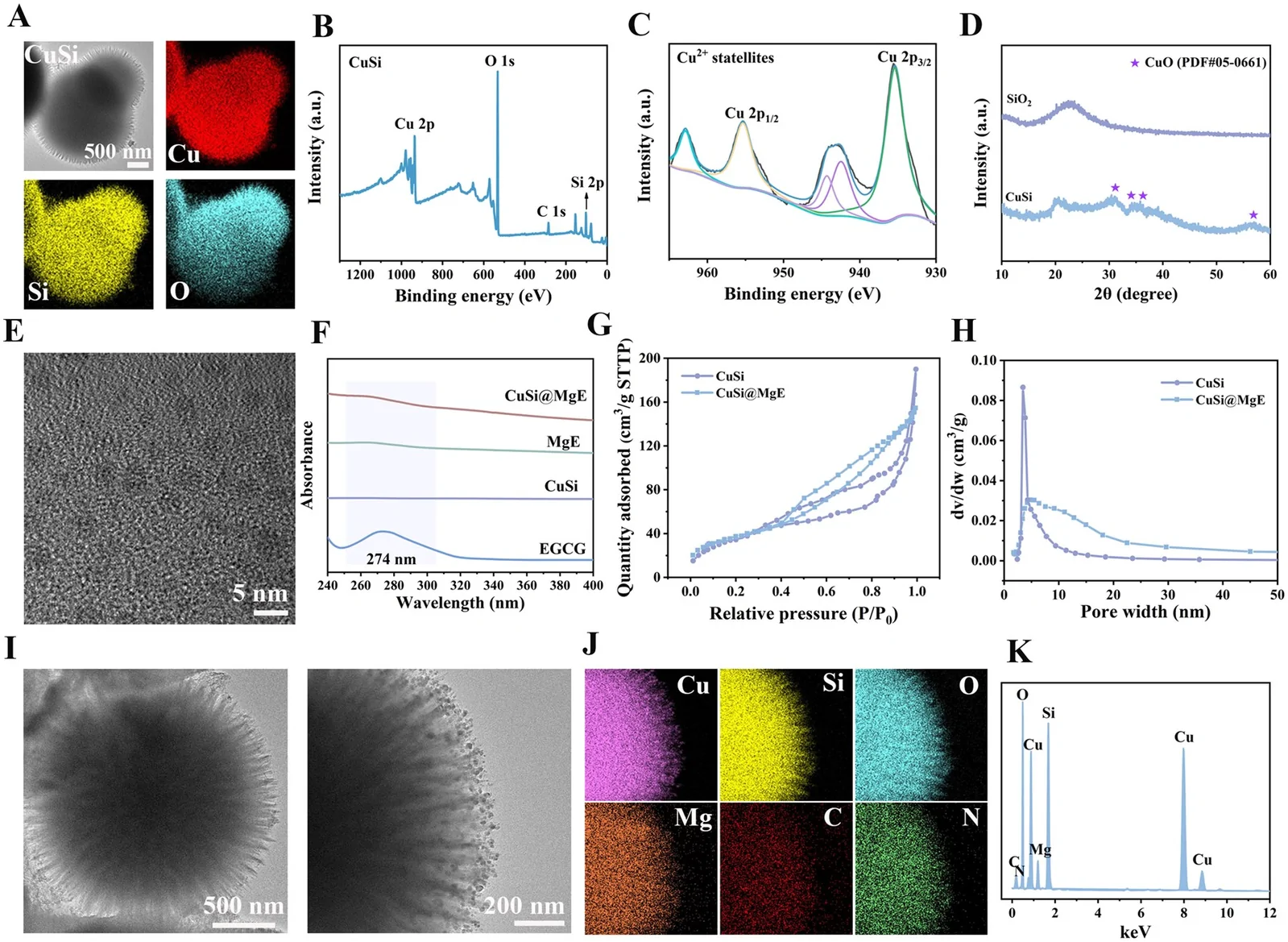
(**A**) TEM image of CuSi nanoparticles and element distribution (Cu, Si and O). (**B**) XPS spectrum of CuSi nanoparticles. (**C**) XPS spectrum of Cu in CuSi nanoparticles. (**D**) XRD spectrum of SiO_2_ and CuSi nanoparticles. (**E**) TEM image of MgE nanoparticles. (**F**) UV absorption spectra of EGCG, CuSi, MgE and CuSi@MgE. (**G**) Nitrogen adsorption and desorption curves of CuSi and CuSi@MgE. (**H**) Pore size distributions of CuSi and CuSi@MgE. (**I**) TEM image of CuSi@MgE and its partial enlarged view. (**J**) Element distribution map of CuSi@MgE (Cu, Si, O, Mg, C and N). (**K**) EDS spectrum of CuSi@MgE.

### Preparation and characterization of TO@CuSi@MgE hydrogel

A functional hydrogel comprising ODA and PTA served as a carrier for CuSi@MgE nanoparticles to facilitate the repair of bone defects. The ^1^H NMR spectrum of OSA revealed the presence of a proton of aldehyde signal at 5.0 ppm (Figure 3A), while the ^1^H NMR spectrum of ODA showed a significantly diminished aldehyde signal and the characteristic proton peak of the benzene ring between 6.0 and 7.0 ppm (Figure 3B). Additionally, the FTIR spectrum (Supplementary Figure S1) demonstrated the formation of a new C = O bond at 1730 cm^−1^ and characteristic peaks for the benzene ring in the range of 950–700 cm^−1^. Taken together, these results verified the effective formation of ODA through Schiff base chemistry involving OSA’s aldehyde functionalities and DA’s amino groups. Furthermore, due to oxidation, the OSA chain broke and the average molecular weight decreased from 340 to 54 kDa. Meanwhile, DA increased the cross-linking of ODA (57 kDa). TA was a small molecule with a five-membered 1,2-dithiolane structure, which underwent partial conversion to TA-Na in the presence of NaHCO_3_, thereby enhancing its water solubility [46]. Additionally, under alkaline conditions, TA efficiently catalyzed the ring-opening of disulfide bonds to form polymers, hydrogen bonds, electrostatic interactions and van der Waals forces, which collectively contribute to the gradual formation of a hydrogel. The process was further enhanced by UV induction, which promoted more extensive ring-opening and the establishment of a more stable hydrogel cross-linking network [47]. The FTIR spectrum of the hydrogel (Figure 3C) revealed a peak at 672 cm^−1^ corresponding to the C-S bond in the TA monomer’s five-membered ring. After ring-opening polymerization, this peak essentially disappeared, indicating successful conversion of a significant portion of the TA into PTA. The characteristic peak of the phenyl ring on DA (at 950 cm^−1^) observed in the TO@CuSi@MgE hydrogel further corroborated the successful synthesis of the TO@CuSi@MgE hydrogel. Moreover, XRD characterization (Figure 3D) revealed that while the TA monomer exhibited crystalline peaks, such peaks were nearly absent in both PTA and TO@CuSi@MgE hydrogels, thereby confirming the amorphous structure of the TO@CuSi@MgE hydrogel.

**Figure 3:**
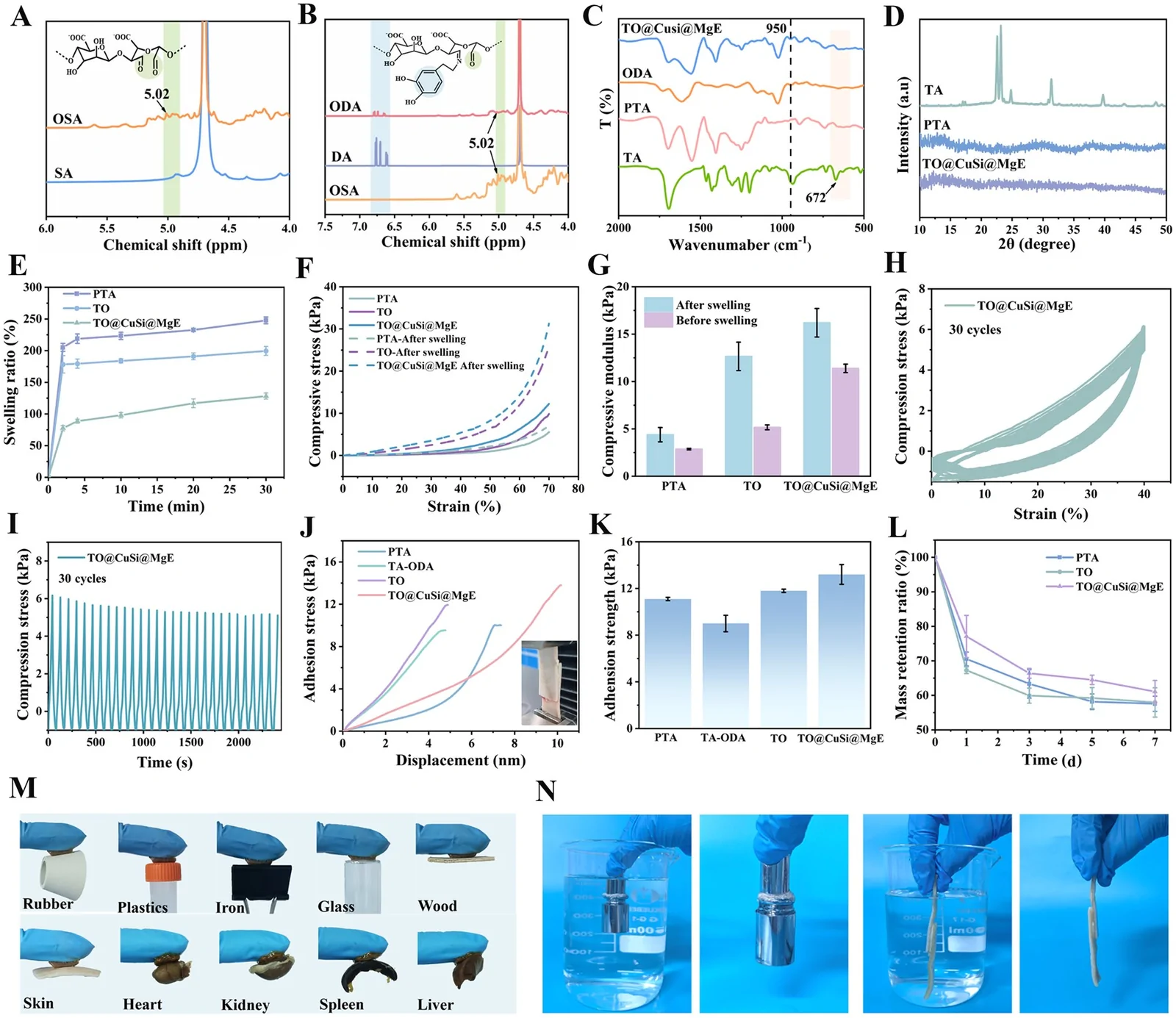
(**A**) Comparative ^1^H NMR analysis of SA and its oxidized form OSA. (**B**) ^1^H NMR profiles of OSA, DA and their reaction product ODA. (**C**) FTIR spectra comparing TA, PTA, ODA and TO@CuSi@MgE. (**D**) X-ray diffraction patterns of TA, PTA and TO@CuSi@MgE. (**E**) Temporal swelling behavior of various hydrogel compositions. (**F**) Mechanical compression profiles of hydrogel samples. (**G**) Calculated compressive moduli of hydrogels. (**H**) Cyclic compression performance (30 cycles at 40% strain) of TO@CuSi@MgE. (**I**) Stress-time relationship during cyclic compression of TO@CuSi@MgE. (**J**) Adhesive interaction profiles between hydrogels and moist porcine skin. (**K**) Quantitative adhesion strength measurements on hydrated porcine skin. (**L**) Degradation remained mass of hydrogels at 1, 3, 5 and 7 days. (**M**) Adhesive testing of TO@CuSi@MgE hydrogel on different substrates. (**N**) Images of TO@CuSi@MgE hydrogel stabilizing metal weights and pig skin in water.

The hydrophobic crosslinked TO@CuSi@MgE hydrogel effectively resisted volumetric swelling in bone repair applications, preserving its mechanical and adhesive properties. Hexyl acrylate’s long alkyl chain and C = C double bond enabled stable PTA crosslinking [48], yielding a low swelling ratio (247 ± 5.35%, Figure 3E). PTA’s carboxyl groups formed hydrogen bonds with DA and SA, further reducing swelling to 199 ± 7.03%. CuSi@MgE nanoparticles strengthened the network, achieving optimal anti-swelling performance (128 ± 4.82%) while maintaining rapid fluid absorption (Supplementary Figure S2). Hydrogel scaffolds must exhibit mechanical compatibility with bone tissue, demonstrating both high elasticity and recovery to withstand intracranial pressure variations while maintaining structural stability during cranial repair. As depicted in Figure 3F, the hydrogels exhibited a high compressive strain, and due to the redistribution of water within the hydrogels during the swelling process, which exhibited higher compressive stress and modulus (Figure 3G). From the SEM images of the freeze-dried scaffolds, it was evident that the PTA scaffold network was simple and loosely structured, whereas TO@CuSi@MgE featured a more intricate and tightly interconnected structure (Supplementary Figure S3). This observation confirmed that the TO@CuSi@MgE hydrogel scaffold showcased superior mechanical properties due to strong hydrogen bonding interactions and the reinforcing effect of CuSi@MgE nanoparticles. To investigate the mechanical stability of the TO@CuSi@MgE hydrogel in wet-state applications, it was immersed in PBS at 37°C for different durations, and the evolution of its volume and mechanical properties was systematically examined (Supplementary Figures S4 and S5a, b). The results showed that the hydrogel exhibited almost no volume swelling, and its mechanical properties gradually increased with prolonged immersion time. This change was mainly attributed to the preferential leaching of free molecules and short-chain oligomers from the hydrogel network, which subsequently induced network reorganization and an increase in crosslinking density. Following this, the TO@CuSi@MgE hydrogel was subjected to 30 cycles of compression at a 40% strain (Figure 3H and Supplementary Figure S6). The cyclic curves exhibited high coincidence and a significant hysteresis loop, indicating the hydrogel’s adaptive energy dissipation when subjected to external forces and its ability to rapidly restore initial stress. In the early cycles, to eliminate stress concentration in the original network, the hydrogel’s stress decreased, but it stabilized in subsequent cycles (Figure 3I). The exceptional elastic recovery capability of the TO@CuSi@MgE hydrogel is attributed to the rich disulfide bonds, hydrogen bonds and other noncovalent dynamic bonds within the network, as well as reversible and dynamically balanced electrostatic interactions, hydrophobic interactions and metal coordination. These interactions facilitate rapid bond rupture and restoration.

In humid environments, water molecules tend to form a hydrated layer on the substrate surface, hindering the close interfacial contact between the hydrogel and the target substrate, consequently resulting in poor adhesion of traditional hydrogel materials in such conditions. Short alkyl chains have been reported to enhance adhesion by excluding water from the interface, thereby exposing adhesive functional groups to interact with the substrate [49]. Here, we ingeniously incorporated hydrophobic alkyl groups onto PTA chains by introducing the crosslinking agent propyl hexanoate, disrupting the hydrated layer and facilitating the exposure of reactive groups. As illustrated in Figure 3J and K, the hydrogel was adhered to wet pigskin, yielding stress–displacement curves. To contrast the effect of hydrophobic chains on adhesion performance, hydrogels without propyl hexanoate (TA-ODA) were prepared, these exhibited the lowest adhesion strength, merely 9.0 ± 0.7 kPa. In comparison, TO, with additional catechol groups and the abundant carboxyl groups on PTA, demonstrated enhanced adhesion. Additionally, the unreacted aldehyde groups on OSA would gradually form Schiff base reactions with amino groups on the tissue. The incorporation of CuSi@MgE nanoparticles increased the cohesive strength of the TO@CuSi@MgE hydrogel, further boosting adhesion strength to 13.8 ± 0.8 kPa. The TO@CuSi@MgE hydrogel demonstrated adhesive properties across various substrates (rubber, plastics, iron, glass and wood), different wet organs of rats (skin, heart, kidney, spleen and liver) (Figure 3M) and rat skull tissue (Supplementary Figure S7). Meanwhile, the TO@CuSi@MgE hydrogel was capable of adhering to a 50 g metal weight and fresh pigskin in water, further substantiating its superior wet adhesion properties (Figure 3N). Additionally, to simulate the wet adhesion effect of the hydrogel in the dynamic bleeding model, a water bottle was vividly used to demonstrate the hydrogel’s ability to seal a damaged bottle, the TO@CuSi@MgE hydrogel maintained a firm closure at the damaged site even under the pressure of squeezing (Supplementary Figure S8). In summary, the excellent wet adhesion of TO@CuSi@MgE hydrogel is attributed to multiple synergistic mechanisms, including interfacial water drainage via hydrophobic interactions, strong hydrogen bonding from hydroxyl and carboxyl groups and dynamic crosslinking through Schiff base bonds. The hydrogel scaffold’s degradation and active substance release affect osteoblast bioactivity, influenced by swelling and environment. EGCG release was tested in acidic/neutral PBS to mimic wound conditions. The CuSi@MgE was subjected to acid hydrolysis using a hydrochloric acid solution at a pH of 2, and the drug-loading capacity of EGCG was calculated to be 62.7 μg/mg based on the standard curve (Supplementary Figure S9). Acidic conditions accelerated CuSi@MgE dissolution (85.75 ± 1.92% release in 70 h, 64.67 ± 1.25% at pH 7.4, Supplementary Figure S10). For cranial repair applications that require long-term stability, the degradation rates of PTA, TO and TO@CuSi@MgE hydrogels were first compared (Figure 3L). Among them, TO@CuSi@MgE exhibited the highest mass retention ratio (61.08 ± 3.22% at 7 days). Subsequently, the degradation behavior of TO@CuSi@MgE was further evaluated over 6 weeks of immersion in PBS (Supplementary Figure S11). The results showed a relatively rapid degradation during the first week, followed by a much slower and stabilized degradation rate thereafter, with a mass retention of 49.97 ± 3.44% at the end of 6 weeks. This degradation profile can be attributed to the initial leaching of oligomers and unpolymerized small molecules from the hydrogel network. The subsequent slow degradation phase ensures that the material maintains its structural integrity and remains effective throughout the required period for cranial bone repair. The safety and effectiveness of the ion release concentrations were evaluated by ICP-OES (Supplementary Figure S12). At week 4, the concentrations of Mg^2+^, Si^4+^ and Cu^2+^ were 6.64 ± 0.08, 15.63 ± 0.19 and 3.69 ± 0.26 μg/mL, respectively, all below the cytotoxic thresholds. These ions contribute to bone regeneration (Mg^2+^ and Si^4+^) and angiogenesis (Cu^2+^), respectively [50–52].

### Antioxidant and antibacterial properties of hydrogels

The presence of cranial defects with open wounds, compounded by oxidative stress and bacterial infections, constitutes a key factor delaying tissue repair. The antioxidant properties of the TO@CuSi@MgE hydrogel were evaluated using the DPPH assay. As shown in Figure 4A and B, PTA, TO and TO@CuSi@MgE hydrogels exhibited negligible absorption peaks at 517 nm, with TO@CuSi@MgE demonstrating a radical scavenging ratio of 85.85 ± 4.14%. Furthermore, all three hydrogels displayed excellent ABTS radical scavenging activity (Figure 4C), with TO@CuSi@MgE achieving a scavenging ratio of 99.24 ± 0.29% (Figure 4D). Hydrogen peroxide scavenging capacity was assessed via the salicylic acid colorimetric reaction with hydroxyl radicals (·OH), in the presence of H_2_O_2_, Fe^2+^ oxidizes to Fe^3+^ while generating ·OH and the resulting Fe^3+^ forms a stable purple complex with salicylic acid. Figure 4E reveals that PTA, TO and TO@CuSi@MgE effectively scavenged H_2_O_2_ and ·OH in the medium, preventing Fe^3+^ generation and consequently showing no colorimetric response. The complex formed between Fe^3+^ and the phenolic hydroxyl (-OH) and carboxyl (-COOH) groups of salicylic acid is detectable at 510 nm, where all three groups exhibited low absorbance (Figure 4F). The superior antioxidant efficacy of TO@CuSi@MgE is attributed to the remarkable reducing capabilities conferred by abundant phenolic hydroxyl groups in DA and EGCG, synergistically enhanced by the reduced thiol groups of TA.

**Figure 4:**
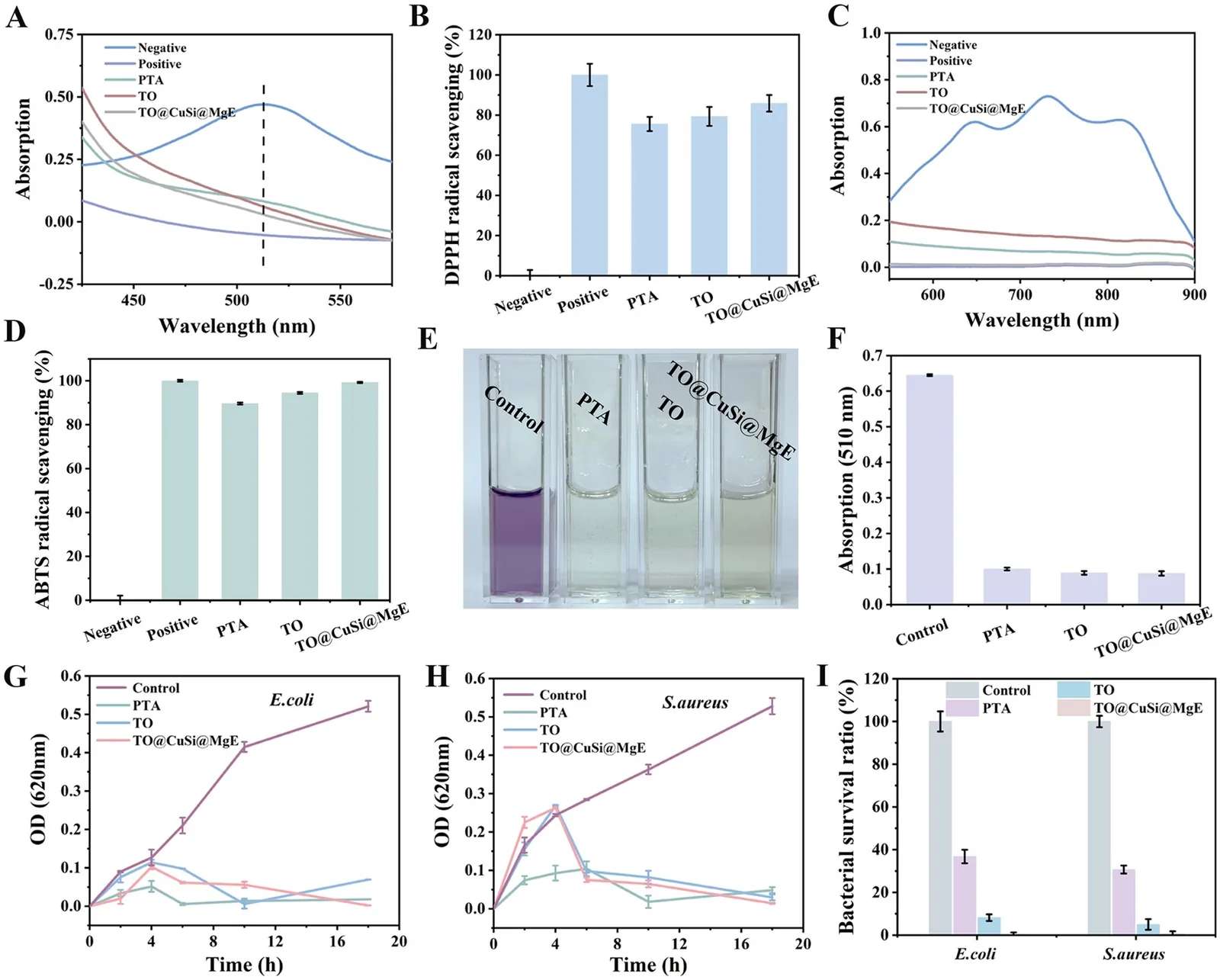
(**A**) DPPH ultraviolet absorption profiles following hydrogel treatments. (**B**) Comparative DPPH radical neutralization capacities across hydrogel compositions. (**C**) ABTS solution absorbance spectra post-hydrogel exposure. (**D**) Quantitative ABTS radical scavenging efficiencies of various hydrogels. (**E**) Visual colorimetric changes in salicylic acid-hydroxyl radical reactions. (**F**) Hydroxyl radical residual levels quantified at 510 nm absorbance. (**G**) *E. coli* proliferation kinetics in hydrogel environments. (**H**) *S. aureus* growth dynamics across hydrogel groups. (**I**) Viable bacterial percentages for both strains following hydrogel treatment.

The antimicrobial properties of TO@CuSi@MgE hydrogel were evaluated against representative Gram-positive (*S. aureus*) and Gram-negative (*E. coli*) bacterial strains. Growth kinetics analysis revealed that while PTA hydrogel showed initial antibacterial effects (attributed to its simple network facilitating rapid active component release), both TO and TO@CuSi@MgE demonstrated sustained antimicrobial activity from 5 h onward (Figure 4G and H). Post 18-h coculture, viable bacterial counts were determined through serial dilution plating (Figure 4I). The TO@CuSi@MgE composite exhibited superior, prolonged antibacterial performance due to synergistic action between Cu^2+^ and EGCG.

### Evaluation of cytocompatibility of hydrogels

The proliferation of MC3T3-E1 cells co-cultured with PTA, TO and TO@CuSi@MgE hydrogels was evaluated using the CCK-8 assay and live/dead cell staining. Significant proliferation was observed after 3 and 5 days of culture, with nearly no red fluorescent dead cells detectable. Compared to the Control, TO and TO@CuSi@MgE groups, the PTA group exhibited lower cell counts, while the introduction of DA into the TO network structure facilitated cell adhesion and proliferation (Figure 5A). The cell viability of all components exceeded 80%, with the TO@CuSi@MgE group achieving a remarkable 97.18 ± 0.85% cell viability on the fifth day (Figure 5B). Subsequently, cellular morphology was observed via CLSM, employing Actin-T and DAPI for staining of the cytoskeleton and nuclei. It is evident from Figure 5C that the TO@CuSi@MgE group shows a fully expanded cell morphology, predominantly exhibiting fusiform and triangular shapes and abundant pseudopodia. The scratch migration assay on the materials can further validate their biocompatibility. As depicted in Figure 5D, compared to the control group, all treatment groups exhibited enhanced cell migration capabilities. Notably, the TO@CuSi@MgE group almost closed the scratch after 48 h, with a migration ratio quantified at 86.26 ± 6.08%, far exceeding that of the TO group (62.14 ± 1.98%) and the PTA group (27.80 ± 4.48%) (Figure 5E). Subsequently, the capacity of materials to induce cellular migration was assessed via transwell experiments. As illustrated in Supplementary Figure S14, the number of migrating cells in the TO@CuSi@MgE group was significantly higher than in the other groups, thereby substantiating the propitious influence of the TO@CuSi@MgE hydrogel on cellular migration. These results collectively suggest that the release and degradation of CuSi@MgE over time, releasing active substances such as EGCG, Mg and Si are conducive to cell proliferation. Additionally, the adhesive groups facilitate cell adhesion, proliferation and migration, while the hydrophobic nature of the hydrogel network forms hydrophobic interactions with the cell membrane, promoting cell aggregation. In summary, the TO@CuSi@MgE hydrogel demonstrates excellent cellular compatibility, which is expected to significantly aid in the repair of bone defects.

**Figure 5:**
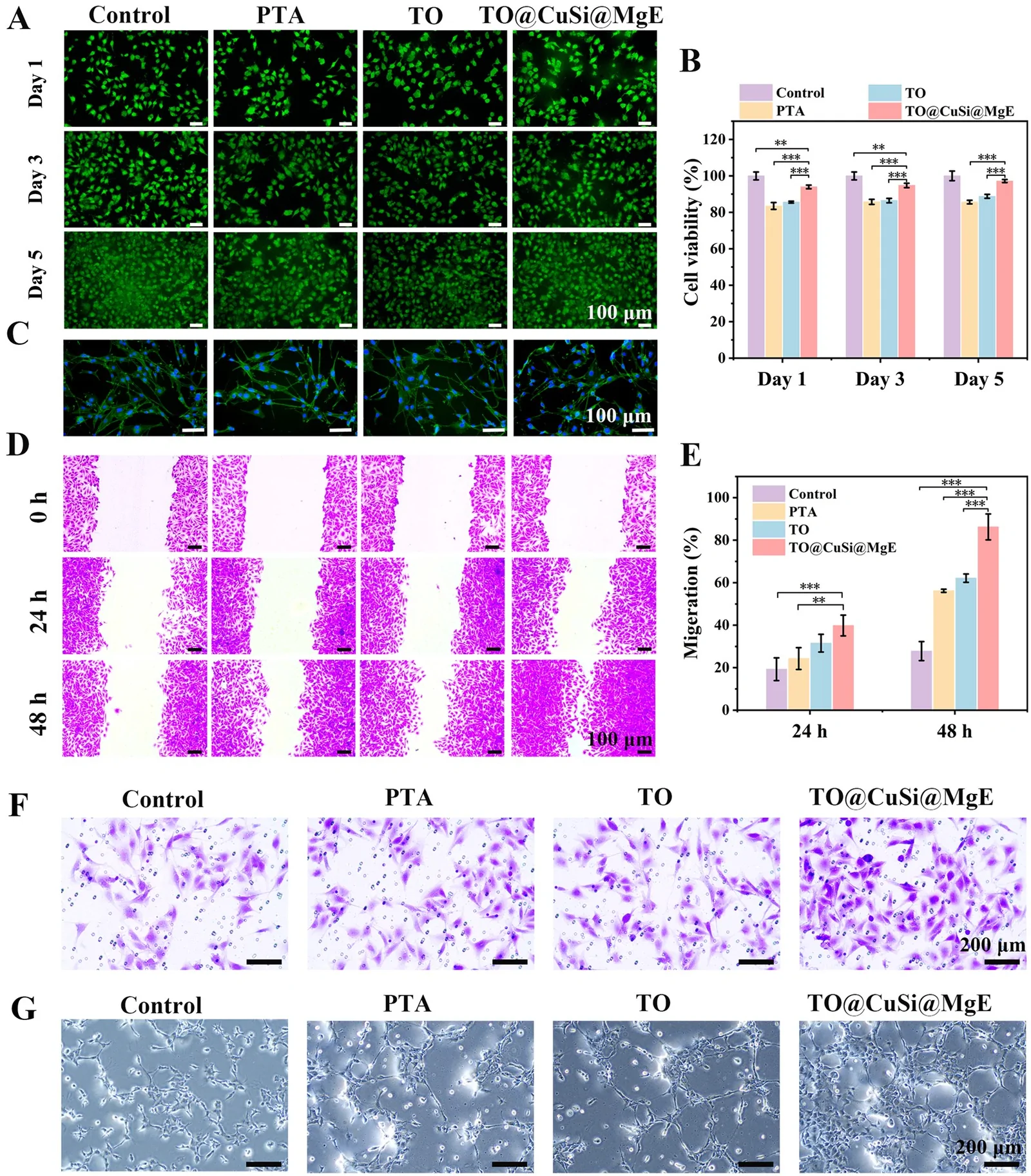
(**A**) Fluorescence micrographs of live/dead stained cells cultured with different hydrogels at 1, 3 and 5-day intervals. (**B**) Quantitative cell viability assessed by CCK-8 assay. (**C**) Confocal microscopy images showing cytoskeletal organization. (**D**) Scratch wound healing assay demonstrating cell migration capacity with various hydrogels and (**E**) statistical analysis of migration ratio. (**F**) Images of transwell migration assay for HUVECs. (**G**) Representative images showing the promotion of HUVEC tube formation by different components. *P < 0.05, **P < 0.01, ***P < 0.001.

During cranial defect repair, rapid neovascularization establishes a network for oxygen and nutrient supply, thereby creating a supportive microenvironment for subsequent osteogenesis [53]. To evaluate the pro-angiogenic capacity of the TO@CuSi@MgE hydrogel, we performed tube formation assays using HUVECs. As shown in Figure 5F and G, the TO@CuSi@MgE group exhibited a substantially higher cell density compared to all other groups. Notably, unlike the control group, which displayed a disrupted network with no closed loops, the TO@CuSi@MgE group formed well-defined tubular structures with multiple branching points and clear lumens, indicating a robust pro-angiogenic effect. Additional tube formation experiments were conducted to confirm the functional attribution of the vasculogenic-promoting ability in the TO@CuSi@MgE system (Supplementary Figure S15). The CuSi group exhibited markedly enhanced tube formation compared with the SiO_2_ group, while the CuSi@MgE group showed no significant difference from the CuSi group, confirming that the major pro‑tube‑formation effect in this system is attributable to Cu^2+^.

### ROS scavenging, macrophage polarization regulation and osteogenic properties

The presence of cranial defects often results in an elevated concentration of ROS, which causes oxidative stress and reduce osteoblast activity or even lead to death, thereby impeding the healing of bone defects. Building upon TA’s established antioxidant capacity, we evaluated hydrogel ROS-scavenging performance using DCFH-DA fluorescence. Figure 6A demonstrates markedly reduced fluorescence in hydrogel-treated samples, particularly in the TO@CuSi@MgE group showing minimal ROS signals. Corresponding live/dead assays (Figure 6B) revealed substantial cell death in H_2_O_2_-treated controls, while TO@CuSi@MgE maintained cell viability with negligible red fluorescence. Quantitative analysis of the live/dead cell ratio (Figure 6C) revealed that the TO@CuSi@MgE hydrogel group had the highest proportion of live cells, indicating the superior ROS-scavenging ability of the TO@CuSi@MgE hydrogel, which effectively protects cells from oxidative stress-induced cell death.

**Figure 6:**
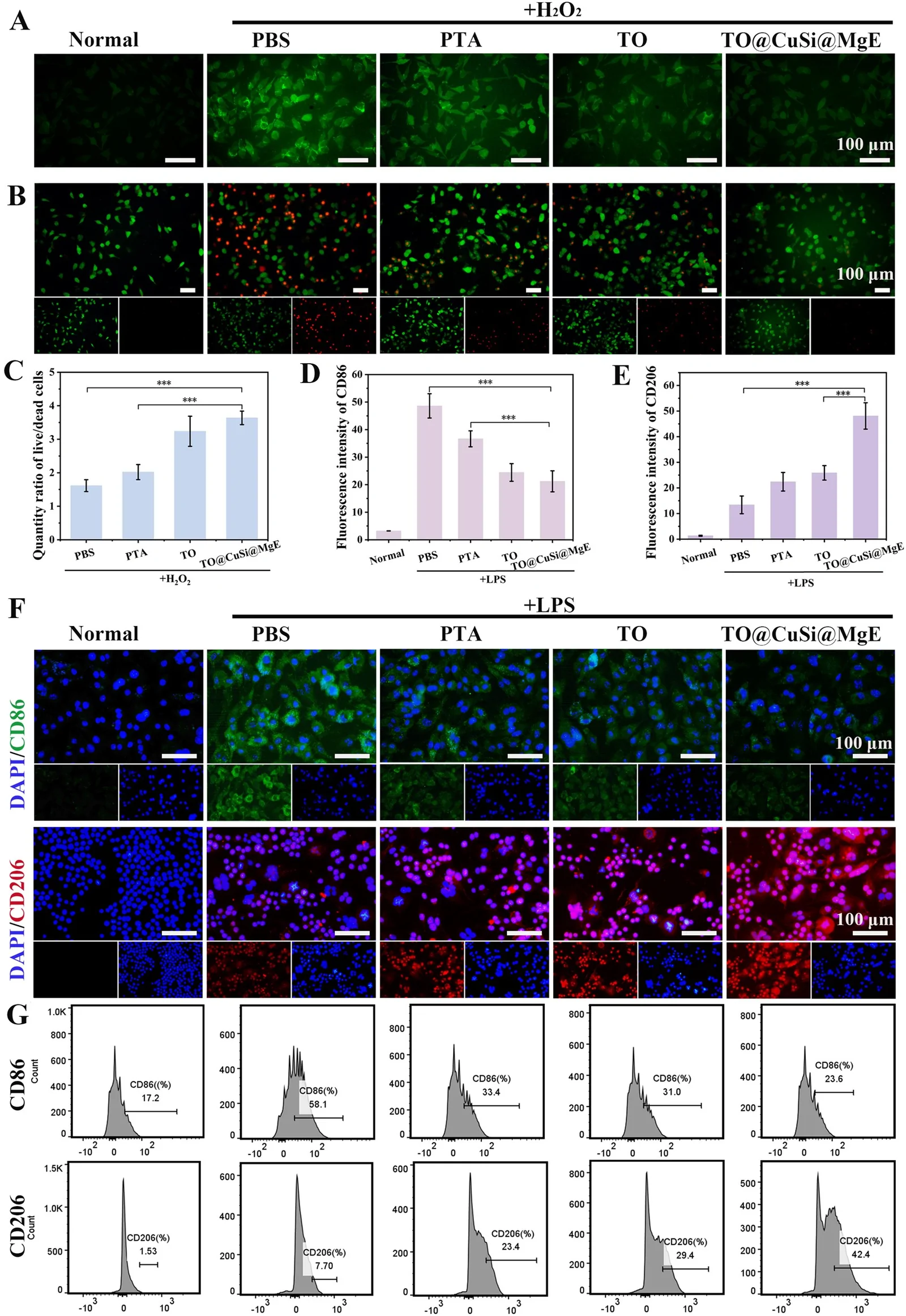
(**A**) DCFH-DA fluorescence staining of ROS in MC3T3-E1. (**B**) Staining images of live and dead cells of ROS in MC3T3-E1 and (**C**) quantitative analysis of the ratio of living/dead cell number. Fluorescent quantitative intensity analysis of (**D**) CD86 and (**E**) CD206. (**F**) Images depicting the expression of CD86 and CD206 in macrophages following co-culture with hydrogels of various compositions. (**G**) Flow cytometry analysis of CD86^+^ macrophages and CD206^+^ macrophages after 48 h treatment with hydrogel. **P < 0.01, ***P < 0.001.

In the context of cranial wound injuries, inflammatory responses are commonly observed, wherein macrophages, as integral components of the innate immune system, engage in host defense, tissue repair and immunoregulation. Among these, M1 macrophages predominantly secrete pro-inflammatory cytokines, perpetuating a chronic inflammatory state that impedes the repair of osseous defects. Consequently, the influence of hydrogels on the polarization of macrophages toward the M1/M2 phenotypes was investigated. Initially, RAW264.7 cells were stimulated with lipopolysaccharide (LPS) to induce the M1 phenotype, followed by co-culturing with TA, TO and TO@CuSi@MgE hydrogels. The levels of CD86 and CD206 in macrophages were, respectively, marked using green and red fluorescence. As illustrated in Figure 6F, the control group, devoid of any treatment, exhibited negligible expression of CD86 and CD206. Conversely, the LPS-treated group displayed high expression of CD86, which gradually decreased with hydrogel treatment, while red fluorescence significantly intensified. Fluorescence quantification (Figure 6D and E) demonstrated TO@CuSi@MgE hydrogels significantly decreased M1 marker (CD86) while increasing M2 marker (CD206) expression, suggesting their ability to promote macrophage polarization from pro-inflammatory M1 to anti-inflammatory M2 phenotype. Flow cytometric analysis (Figure 6G) confirmed that TO@CuSi@MgE hydrogels yielded the highest M2 macrophage percentage and lowest M1 population versus TO hydrogels, ultimately enhancing the M2/M1 ratio. This further demonstrates that the MgE metal polyphenol has a direct effect in promoting the polarization of macrophages (Supplementary Figure S16). This is because the metal-polyphenol network significantly enhances the loading capacity and stability of EGCG in the material and also strengthens its immunomodulatory function [54].

To unveil the underlying mechanisms by which the TO@CuSi@MgE hydrogel modulates osteogenic differentiation, genes pertaining to cranial defect and osteogenic function were procured from GeneCards, a comparative analysis revealed 1399 overlapping genes (Figure 7A). Functional enrichment analysis of the overlapping genes through GO classification demonstrated significant involvement in biological processes related to bone formation, cellular proliferation and motility (Supplementary Figure S17). These findings imply that TO@CuSi@MgE hydrogel could promote calvarial regeneration by stimulating cell expansion and migration. KEGG pathway analysis further identified the PI3K-AKT signaling cascade as a key regulator of osteogenic differentiation and cranial repair (Figure 7B). Therefore, we hypothesized that TO@CuSi@MgE hydrogel might promote osteogenesis by activating PI3K/AKT signaling pathway, this signaling pathway is pivotal in intracellular regulation of cell proliferation, differentiation and migration. RT-qPCR assays demonstrated a notable increase in the expression of PI3K, AKT and mTOR following treatment with TO@CuSi@MgE (Figure 7C), confirming that TO@CuSi@MgE hydrogel scaffold can activate PI3K/AKT signaling pathway to stimulate osteogenic differentiation.

**Figure 7:**
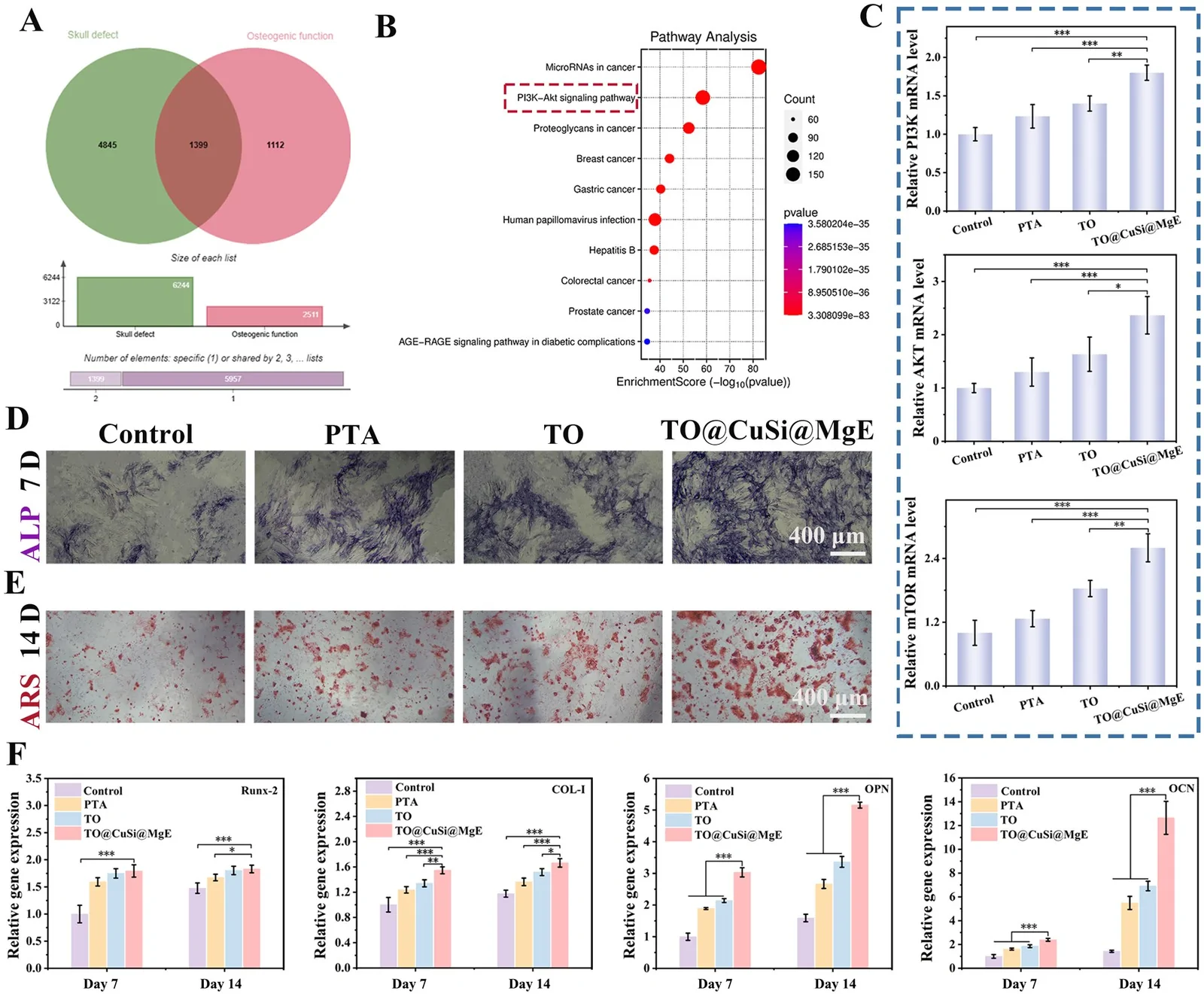
(**A**) Venn diagrams showing genes associated with skull defect and osteogenic function obtained from GeneCards. (**B**) KEGG analysis of duplicate genes. (**C**) Expression levels of PI3K, AKT and mTOR. Staining analysis of (**D**) ALP and (**E**) ARS. (**F**) Expression levels of osteogenic genes (Runx-2, COL-I, OPN and OCN) in cells co-cultured on hydrogels of different compositions over 7 and 14 days. *P < 0.05, **P < 0.01, ***P < 0.001.

The efficacy of TO@CuSi@MgE hydrogel osteogenic activity *in vitro* was determined. During the process of bone regeneration, the formation of biominerals such as Mg^2+^ plays a crucial role. Initially, osteoblasts synthesize key components of the extracellular matrix, such as COL-I, OCN and OPN. Subsequently, under the catalytic influence of alkaline phosphatase (ALP), calcium phosphate precipitates onto collagen fibrils, thereby promoting the mineralization of the matrix and ultimately resulting in the formation of bone tissue. As a marker of early osteogenic differentiation, ALP activity was assessed in grown on various hydrogels for seven days. From Figure 7D, it is evident that after 7 days of culture, the TO@CuSi@MgE hydrogel exhibited the highest ALP activity. Additionally, following 14 days of co-cultivation and the implementation of ARS, the TO@CuSi@MgE hydrogel group displayed more extensive and abundant red mineralized nodules compared to the other groups (Figure 7E). Quantitative analysis of ALP and ARS confirmed the visual trends observed (Supplementary Figures S18 and S19). To delve into the underlying molecular mechanisms, osteogenic-related genes (Runx-2, COL-I, OPN and OCN) in mRNA expression were examined using the RT-qPCR technique (Figure 7F). According to the relative expression patterns of these genes in different hydrogel groups over 7 and 14 days, it is evident that the osteogenic gene expression in the TO@CuSi@MgE hydrogel group significantly surpasses that of the other groups. Specifically, the levels of Runx-2 remain relatively stable during both the 7th and 14th days, indicating a sustained signaling demand for Runx-2 by the cells. Additionally, COL-I, OPN and OCN demonstrate a progressive increase over time, potentially fulfilling the requirements for bone formation and mineralization. The TO@CuSi@MgE hydrogel exhibits excellent osteogenic capability, which can be attributed to the following synergistic mechanisms: the early immunomodulatory effect creates a favorable microenvironment for cell growth, the sustained release of Cu^2+^ not only avoids ion toxicity but also continuously stimulates angiogenesis, providing sufficient nutrients for osteogenesis. Meanwhile, Mg^2+^ and Si^4+^ further induce osteogenic differentiation, thereby accelerating bone regeneration. These findings provide data support for the subsequent *in vivo* application of this material.

### Evaluation of *in vivo* skull defect repair ability

To investigate the efficacy of TO@CuSi@MgE hydrogel in repairing bone defects *in vivo*, a rat cranial defect model (diameter 5 mm) was established, and the healing process was observed over a period of 14 weeks. Comprehensive analysis of new bone formation induced by the hydrogel scaffold was conducted via micro-computed tomography (micro-CT). As shown in Figure 8A, while the control group still exhibited a significant cranial defect, all hydrogel-treated groups displayed varying degrees of new bone formation, with the TO@CuSi@MgE hydrogel group showing extensive bone expansion toward the center of the bone cavity, exhibiting a relatively intact bone structure. Quantitative analysis of bone morphometric parameters (BV/TV, Tb.N, Tb.Th and Tb.Sp) demonstrated superior bone formation and well-developed microarchitecture in the TO@CuSi@MgE hydrogel group compared to other treatments (Figure 8B). The osteogenic efficiency of TO@CuSi@MgE hydrogel is significantly higher than that of rigid repair materials (with BV/TV lower than 20%) [55] and photoinitiated *in situ* adhesive hydrogel (with BV/TV about 15%) [56], indicating that the TO@CuSi@MgE hydrogel is more conducive to inducing bone regeneration. Furthermore, the H&E staining results of the internal organs of the rats showed no obvious inflammation, indicating that the TO@CuSi@MgE hydrogel has no long-term toxicity *in vivo* (Supplementary Figure S20).

**Figure 8:**
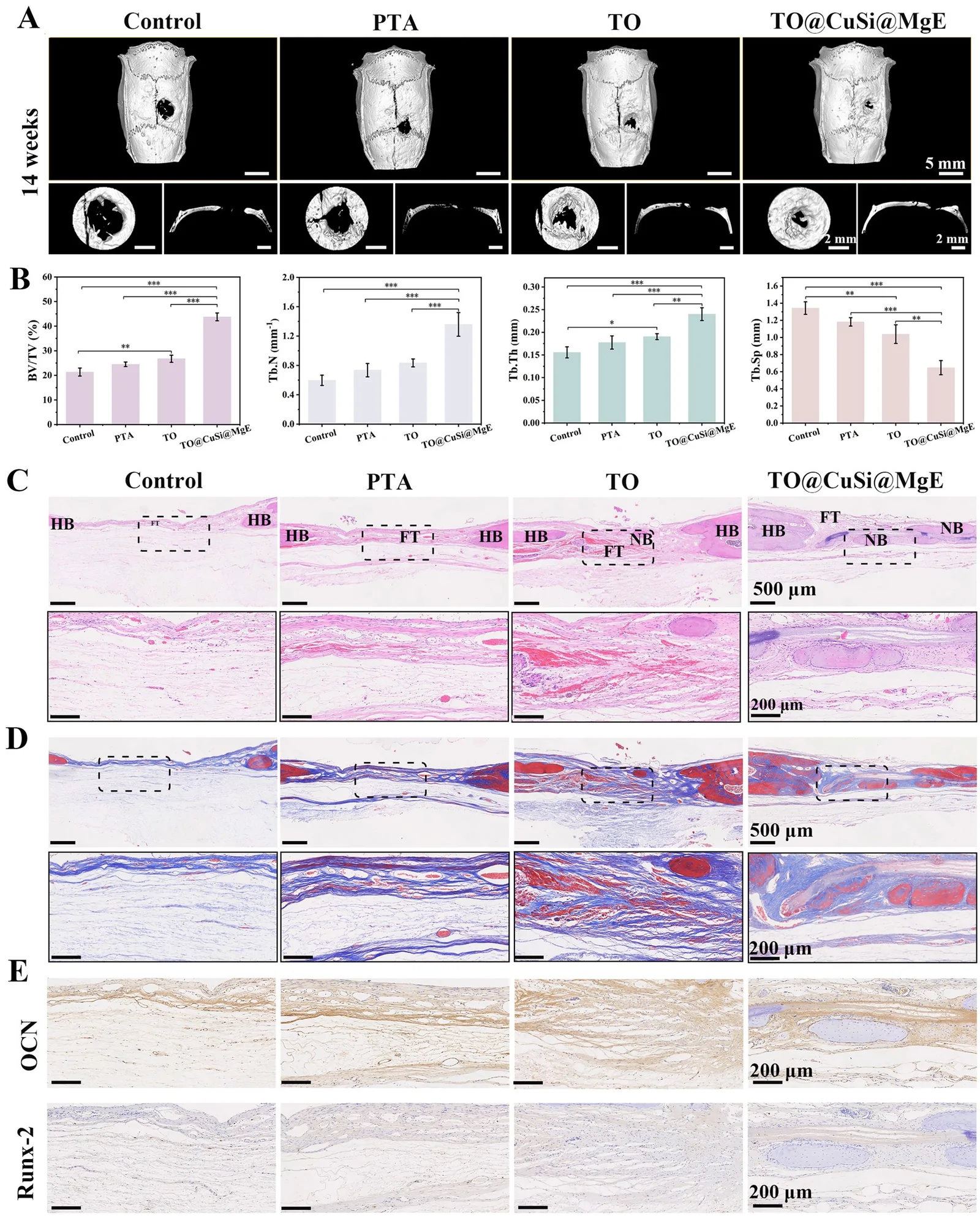
(**A**) Three-dimensional micro-CT reconstructions of cranial bone defects 14 weeks post-implantation with different scaffold groups, with corresponding (**B**) quantitative morphometric analysis including BV/TV, Tb.N, Tb.Th and Tb.Sp parameters. (**C**) Hematoxylin-eosin and (**D**) Masson’s trichrome stained sections with insets showing higher magnification views. (**E**) Immunohistochemical localization of OCN and Runx-2 at the 14-week time point. **P < 0.01, ***P < 0.001. NB: new bone; FT: fibrous tissue; HB: host bone.

Subsequent histomorphometry analysis of the regenerated bone revealed that, as evidenced by hematoxylin and eosin (H&E) staining (Figure 8C), no discernible deposition of bone matrix was observed in the 14-week blank group. In contrast, the regenerated bone in the TO@CuSi@MgE hydrogel group exhibited close integration with the host bone, with mature bone matrix, abundant and dense bone tissue and the defect area was almost entirely covered, displaying a relatively intact lamellar structure similar to that of normal cranial bone. Masson staining, employed to visualize collagen fibers and collagen production (Figure 8D), demonstrated significant collagen deposition at the site of bone defects in the TO@CuSi@MgE hydrogel group, with mature collagen (depicted in red areas) and collagen fibers (shown in blue areas) being clearly observed. Immunohistochemical staining was further utilized to assess the expression levels of osteogenic-related proteins (OCN and Runx-2), validating the generation of newly formed bone tissue (Figure 8E). The TO@CuSi@MgE hydrogel group exhibited high expression levels of both OCN and Runx-2, followed by TO and PTA, respectively, consistent with the results obtained from micro-CT imaging and histomorphometry analysis.

Macrophage polarization toward the M2 phenotype accelerates bone defect repair by promoting transition from inflammatory responses to tissue regeneration processes. Immunofluorescence staining of CD86 and CD206 in defect areas at 14 weeks postoperation was performed (Figure 9A), followed by quantitative analysis of fluorescence signals (Figure 9D and E). The control group still exhibited significant red fluorescence, indicating the presence of numerous M1 phenotype macrophages in the wound tissue, reflective of a certain degree of inflammatory response. Conversely, the hydrogel-treated groups showed stronger green fluorescence, with both TO and TO@CuSi@MgE hydrogels demonstrating a reduction in the number of M1 and M2 phenotype macrophages, suggesting that tissue repair had progressed from the inflammatory phase to tissue remodeling, achieving a relative balance and stability between M1 and M2 macrophages. Bone regeneration requires robust vascular support; therefore, immunofluorescence labeling using CD31 and α-SMA was applied to the cranial tissue samples at the 14th week to assess potential angiogenesis (Figure 9B and C). The TO@CuSi@MgE hydrogel group exhibited a higher vascular density and maturity, with the quantitative analysis of CD31 and α-SMA fluorescence showing consistent results (Figure 9F and G). In summary, the TO@CuSi@MgE hydrogel exhibits a high efficiency in promoting cranial bone defect repair. This is attributed to the TO@CuSi@MgE hydrogel’s ability to modulate an osteogenic microenvironment conducive to bone formation by inducing macrophage M2 polarization, enhancing vascularization and bolstering osteogenesis.

**Figure 9:**
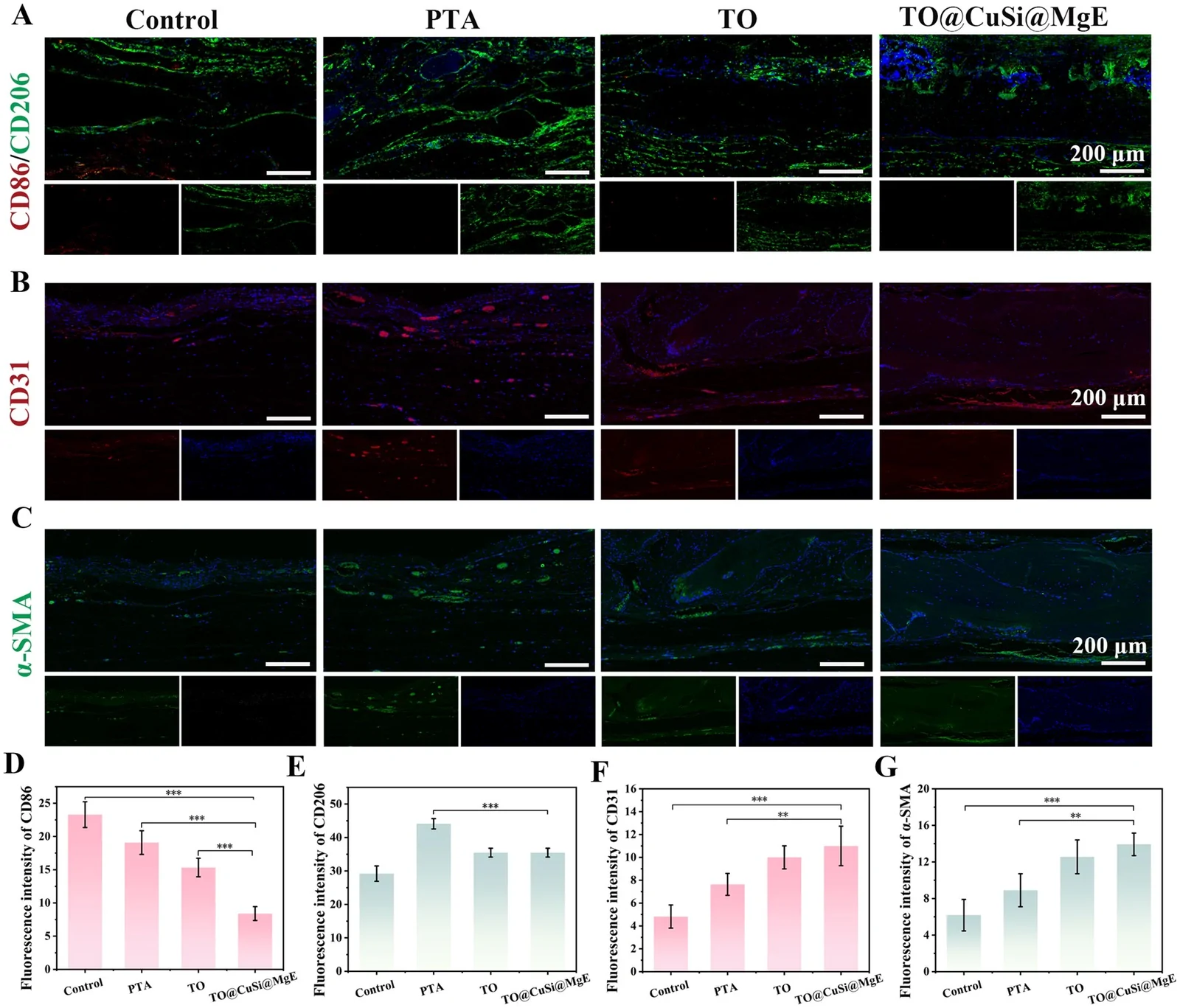
(**A**) Representative immunofluorescence micrographs showing CD86 and CD206 co-staining in calvarial defect sections at 14 weeks. (**B**) Fluorescence visualization of CD31 expression. (**C**) α-SMA fluorescent labeling patterns. (**D, E**) Quantitative intensity measurements of CD86 and CD206 signals. (**F, G**) Statistical analysis of CD31 and α-SMA fluorescence intensities. *P < 0.05, **P < 0.01, ***P < 0.001.

## Conclusion

To overcome the dual challenges of pathological microenvironment dysregulation and compromised osseointegration in cranial defect healing, we developed an integrated TO@CuSi@MgE hydrogel scaffold featuring multistage functional repair and a strong wet adhesion property. The composite simultaneously remodels the osteoimmune microenvironment via potent ROS scavenging, antibacterial activity and macrophage M2 polarization while promoting cell proliferation, angiogenesis and osteogenic differentiation. *In vivo* validation demonstrates significantly accelerated cranial defect regeneration. This integrated strategy pioneers a biomimetic platform that concurrently addresses microenvironmental imbalances and osseointegration barriers, representing a critical advancement toward next-generation bone regenerative therapies.

## Supplementary Material

rbag183_Supplementary_Data

## Data Availability

Data will be made available on request.
