# Supplementary material for: Multistage functional nanoplatform synergizing with robust wet-adhesive scaffolds for accelerated cranial bone regeneration
Source: Regen Biomater. 2026 Sep 10;13:rbag183. doi: 10.1093/rb/rbag183 (PMC13722442; doi:10.1093/rb/rbag183)
Supplement: Supplementary file 1 [file rbag183_supplementary_data.docx]

# Multi-stage Functional Nanoplatform Synergizing with Robust Wet-Adhesive Scaffolds for Accelerated Cranial Bone Regeneration

Xiaohua Su^a, b, 1^, Nashun bayaer^c, 1^, Xi Long^d, 1^, Qi Li^c^, Siying Liu^e^, Siying Cheng^f^, Weiquan Shen^e^, Wenqiang Li^g,^ *, Yi Zhan^h,^ *

^a^ Clinical Research Center, The First Dongguan Affiliated Hospital, Guangdong Medical University, Dongguan, Guangdong, 523710, China

^b^ Laboratory Animal Center, Guangdong Medical University, Dongguan, Guangdong, 523808, China

^c^ School of Basic Medical Sciences, Inner Mongolia Medical University, Chilechuan Dairy Economic Development Zone, Hohhot, 010110, China

^d^ The Eighth People's Hospital of Nanhai District, Foshan, Guangdong, 528216, China

^e^ College of Pharmacy, Guangdong Medical University, Dongguan, Guangdong, 523808, China

^f^ College of medical technology, Guangdong Medical University, Dongguan, Guangdong, 523808, China

^g^ The Affiliated Guangdong Second Provincial General Hospital of Jinan University, Guangzhou, Guangdong, 510315, China

h Department of Orthopedic, The First Dongguan Affiliated Hospital, Guangdong Medical University, Dongguan, Guangdong, 523710, China

^1^ These authors contributed equally to this work.

*Corresponding authors.

E-mail addresses: 405119923@qq.com (W. Li), zhanyi172@126.com (Y. Zhan).

**Figure S1** FT-IR spectrums of DA, SA and ODA.

**Figure S2** Digital photo of hydrogels before and after swelling.

**Figure S3** SEM images of PTA and TO@CuSi@MgE.

**Figure S4** Photographs of the TO@CuSi@MgE hydrogel immersed in PBS for different numbers of days.

**Figure S5** (a) The compression stress-strain curves and (b) compression modulus of the TO@CuSi@MgE hydrogel after being immersed in PBS for different periods of time.

**Figure S6** Digital photos of TO@CuSi@MgE hydrogels with compression 40% strain.

**Figure S7** Photo of rat skull adhesive TO@CuSi@MgE hydrogel.

**Figure S8** Image of TO@Cusi@MgE hydrogel seals the bottle with damaged

**Figure S9** The standard curve of EGCG in HCl solution with a pH of 2.

**Figure S10** Release of EGCG in PBS at different pH values.

**Figure S11** The mass retention rate of TO@Cusi@MgE hydrogel after 6 weeks of degradation.

**Figure S12** The cumulative ion release curve of TO@Cusi@MgE hydrogel over a period of 4 weeks

**Figure S13** Photographs of coated plates after 18 h of co-cultivation of different component hydrogels with *E. coli* and *S. aureus*.

**Figure S14** Images of Transwell invasion assay at different components.

**Figure S15** Representative images showing the promotion of HUVEC tube formation by different components.

**Figure S16** Images depicting the expression of CD86 (green) and CD206 (red) in macrophages following co-culture with various compositions.

**Figure S17** GO enrichment analysis of duplicate genes.

**Figure S18** Activity of ALP.

**Figure S19** OD values of ARS.

**Figure S20** H&E staining images of major organs of rats.
